# Multi-compartment omics identifies a reproducible association between circulating 5-androstene-3β,17β-diol and mastitis clinical status in dairy cows

**DOI:** 10.3389/fmicb.2026.1955642

**Published:** 2026-09-30

**Authors:** Yuqiong Li, Haihui Gao, Zhen Dong, Jian Gao, Liang Huang, Song Hua, Zhuming Zhang, Hongbing Li, Chenglian Zhang, Meizhou Huang

**Affiliations:** 1 Institute of Animal Science, Ningxia Academy of Agricultural and Forestry Sciences, Yinchuan, China; 2 Hunan Province Key Laboratory of Traditional Chinese Veterinary Medicine, Hunan Agricultural University, Changsha, China; 3 College of Veterinary Medicine, China Agricultural University, Beijing, China; 4 Ningxia Agricultural Land Reclamation Changshan Tou Farm Company Limited, Yinchuan, China; 5 College of Veterinary Medicine, Northwest A&F University, Xianyang, China; 6 College of Animal Science and Technology, Ningxia University, Yinchuan, China; 7 Ningxia Hui Autonomous Region Animal Disease Prevention and Control Center, Yinchuan, China; 8 Metabolic Hepatobiliary and Pancreatic Diseases Key Laboratory of Luzhou City, Academician (Expert) Workstation of Sichuan Province, Department of General Surgery (Hepatopancreatobiliary Surgery), The Affiliated Hospital of Southwest Medical University, Luzhou, China

**Keywords:** blood–milk barrier, complement activation, Holstein cow, rumen microbiota, steroid metabolism

## Abstract

**Introduction:**

Bovine mastitis is generally viewed as a localized intramammary infection, but the extent to which gastrointestinal, circulating, and mammary tissue features covary across clinical phenotypes remains unclear. We aimed to characterize concurrent alterations across the rumen, hindgut, serum, milk, and mammary gland in healthy control (HC), subclinical mastitis (SCM), and clinical mastitis (CM) cows and to evaluate circulating 5-androstene-3β,17β-diol (5-AED) as a correlate of mastitis phenotype.

**Methods:**

In a discovery cohort, fecal and rumen microbiota were characterized by 16S rRNA gene sequencing, and untargeted metabolomics was performed across four biological matrices. Mammary tissue was analyzed using tandem mass tag (TMT)-based proteomics, parallel reaction monitoring (PRM), and histology, while circulating inflammatory and metabolic mediators were measured in serum. A later cohort from the same herd was analyzed independently to replicate the serum 5-AED findings using targeted analysis.

**Results:**

Systemic inflammatory and lipid mediators differed in SCM cows despite the absence of overt clinical signs. Fecal and rumen microbial community structures differed among HC, SCM, and CM groups, with lower rumen microbial richness in mastitis cows. 16S rRNA-based functional predictions indicated differences in carbohydrate- and amino acid-related functional potential. Serum 5-AED showed the highest distribution in HC, an intermediate distribution in SCM, and the lowest distribution in CM, and this group ordering was reproduced in the later cohort. Mammary tissue exhibited lower abundance of claudin-8, fatty acid synthase, acetyl-CoA carboxylase, and ABCG2, together with enrichment of complement and coagulation pathways and annotation of a neutrophil extracellular trap-associated pathway. Targeted proteomics supported the direction of selected barrier-, lipid-, complement-, and fibrinogen-related differences. Hydroxysteroid 17β-dehydrogenase abundance also differed among groups; however, enzyme activity and a direct biochemical relationship with 5-AED were not established.

**Discussion:**

These findings reveal coordinated differences across gastrointestinal, circulating, and mammary compartments associated with distinct mastitis phenotypes, including subclinical disease. However, the cross-sectional design does not establish temporal progression, mastitis specificity, or a causal gut–mammary pathway. The association between circulating 5-AED and mastitis phenotype warrants prospective evaluation in independent herds using an independently validated analytical assay before its potential clinical utility can be assessed.

## 1 Introduction

Bovine mastitis is initiated most commonly by pathogens entering through the teat canal, yet disease expression is shaped by the host’s metabolic and immune state (Ruegg, 2017; Dervishi et al., 2015). Subclinical mastitis (SCM) is defined by increased somatic cell count in the absence of visible udder or milk abnormalities, whereas clinical mastitis (CM) includes overt local signs and abnormal milk. These categories are clinically useful but should not be assumed to represent a fixed temporal sequence. Their divergent tissue and systemic phenotypes nevertheless suggest that processes outside the local mammary compartment may contribute to heterogeneity across clinical phenotypes (Lisuzzo et al., 2026).

Experimental and observational studies have implicated gut and rumen microbial communities in mammary inflammation, giving rise to the gut/rumen-mammary axis concept (Hu et al., 2020, 2024). However, most evidence remains associative, and changes in feed intake, inflammation, and antimicrobial exposure can themselves reshape gastrointestinal communities. The molecular features that covary across the gastrointestinal tract, circulation, and mammary gland in naturally occurring bovine mastitis therefore remain incompletely resolved (Ibeagha-Awemu et al., 2025). A multi-compartment design can identify reproducible cross-organ signatures while avoiding claims of directionality that require longitudinal or transfer experiments.

Two processes are of particular interest. First, sustained neutrophil activation can amplify tissue injury and couple extracellular-trap formation to coagulation (Nathan, 2002; Serhan and Savill, 2005; Greenlee-Wacker, 2016; Xie et al., 2021). Second, endogenous steroid metabolites may modulate inflammatory tone. 5-Androstene-3β,17β-diol (5-AED) has shown anti-inflammatory activity in experimental models, including suppression of Toll-like receptor 4-nuclear factor kappa B signaling (Abbas and Hassan, 2022). Hydroxysteroid 17β-dehydrogenases catalyze substrate- and cofactor-dependent steroid redox reactions, but their relationship to circulating 5-AED in bovine mammary disease has not been established (Hiltunen et al., 2019).

We therefore conducted an observational, cross-sectional study spanning five biological matrices: feces, rumen fluid, serum, milk, and mammary tissue. We hypothesized that mastitis clinical status would be associated with concurrent gastrointestinal microbial, systemic metabolic, and mammary proteomic signatures, and that circulating 5-AED would show a reproducible association with clinical phenotype. The objective was to define these cross-compartment associations and generate testable, rather than presumptively causal, hypotheses for future longitudinal and interventional studies (Guo et al., 2023).

## 2 Materials and methods

### 2.1 Study design

We used an observational, cross-sectional, two-cohort design. Cows were enrolled during two non-overlapping periods at the same commercial dairy: a discovery cohort from March to May 2023 and a same-herd temporal replication cohort from September to November 2023. The later cohort was used for targeted serum 5-AED measurement only and was not an external-herd validation cohort. We initially enrolled 10 cows per group in the discovery cohort and 18 per group in the temporal replication cohort. Between screening and sample collection, six cows developed concurrent non-mammary infections requiring systemic antibiotics and were excluded before laboratory analysis. Final sample sizes were HC *n* = 10, SCM *n* = 9, and CM *n* = 9 in the discovery cohort and HC *n* = 18, SCM *n* = 16, and CM *n* = 16 in the temporal replication cohort. The individual cow was the experimental unit, and the cohorts were analyzed separately. Histology, serum inflammatory and lipid mediators, progesterone, fecal and rumen 16S sequencing, and four-matrix untargeted metabolomics were assigned to the discovery cohort. TMT proteomics and PRM used discovery-cohort mammary-tissue subsets. The later cohort underwent targeted serum 5-AED radioimmunoassay.

### 2.2 Animal selection and sampling

All cows were recruited from one large commercial dairy to reduce environmental and management heterogeneity. Eligible animals were multiparous Holstein cows (parity 2 or 3) at 90–120 days in milk and were housed in free-stall barns. The available herd feeding record described the lactating-cow TMR as 48.1% dry matter, with a reference dry-matter intake of 23.2 kg/cow/day (Table 1). The particle-size targets for the lactating-cow TMR were 4–8% retained on the 19-mm sieve, 30–50% retained on the 8-mm sieve, and 40–60% in the bottom pan. These percentages were farm management targets rather than group-specific or batch-specific analytical measurements. Group-level farm records showed mean dry-matter intakes of 23.8, 19.1, and 17.8 kg/cow/day for HC, SCM, and CM, respectively (Table 1). Corresponding mean milk yields were 38.2, 28.4, and 23.4 kg/cow/day, and mean body-condition scores were 3.18, 3.25, and 3.28 (Table 1). Complete ingredient-level and nutrient-composition records were not available for both enrollment periods, so identical ration composition across periods cannot be confirmed. Cows were excluded if they had a concurrent metabolic disorder, including ketosis or hypocalcemia, or had received systemic antimicrobial or anti-inflammatory treatment during the preceding 30 days.

**Table 1:** Available production, feeding-management, and reproductive baseline information.

Variable	HC	SCM	CM
Parity	2–3	2–3	2–3
Days in milk	90–120	90–120	90–120
Milk yield, kg/cow/day	38.2 ± 1.4	28.4 ± 4.5	23.4 ± 5.7
Dry-matter intake, kg/cow/day	23.8	19.1	17.8
Body-condition score	3.18 ± 0.22	3.25 ± 0.31	3.28 ± 0.24
TMR dry matter, %	48.1	48.1	48.1
TMR retained on 19-mm sieve, %	4–8	4–8	4–8
TMR retained on 8-mm sieve, %	30–50	30–50	30–50
TMR in bottom pan, %	40–60	40–60	40–60

TMR particle-size percentages are farm management targets for the lactating-cow ration rather than group-specific analytical measurements.

Group status was assigned from veterinary examination, quarter-level California Mastitis Test results, and somatic cell count records. HC cows had clinically normal udders, a negative California Mastitis Test, and a somatic cell count <200,000 cells/ml. SCM cows had a positive California Mastitis Test and a somatic cell count >500,000 cells/ml without visible udder inflammation or milk abnormality. CM cows had a positive California Mastitis Test and a somatic cell count >500,000 cells/ml, and at least one local inflammatory sign, such as erythema, edema, or increased udder temperature, together with visible milk changes, including flakes or a watery appearance. Bacteriological culture and molecular pathogen identification were not available for etiological classification. HC, SCM, and CM are therefore interpreted as clinical/SCC-defined phenotypes rather than microbiologically confirmed intramammary infections. A standardized numerical clinical-mastitis score was also unavailable.

Samples were collected between 06.00 and 08.00 h, approximately 1 h before morning feeding. Rumen fluid was obtained by esophageal intubation; the first 20 mL was discarded, and the remaining fluid was passed through four layers of sterile cheesecloth. Feces were collected from the rectum with a new sterile sleeve for each cow. Blood was collected from the coccygeal vein into serum tubes, allowed to clot for 30 min, and centrifuged at 3,000×*g* for 15 min at 4 °C. After teat cleaning with 70% ethanol and foremilk removal, midstream milk was collected aseptically. Mammary core biopsy was taken from a quarter positive on the quarter-level California Mastitis Test in SCM cows, from a quarter showing local and milk abnormalities in CM cows, and from the left-front quarter in HC cows. Biopsies were obtained under ultrasonographic guidance with a 14-gauge needle and local 2% lidocaine anesthesia. When more than one quarter met the relevant criterion, the retained record did not document a standardized selection hierarchy. Quarter location was not included as an analytical covariate.

### 2.3 Histological and biochemical analyses

Mammary tissue was fixed in 4% paraformaldehyde in phosphate-buffered saline (pH 7.4) for 24 h at 4 °C, paraffin embedded, sectioned at 4 μm, and stained with hematoxylin and eosin. Two pathologists independently scored coded sections for alveolar structural damage (0–3), inflammatory-cell infiltration (0–3), and interstitial fibrosis (0–3). The three components were summed to give a total score from 0 to 9. Scores differing by more than one point were resolved by joint review.

In the discovery cohort, serum tumor necrosis factor alpha (TNF-α), interleukin-1 beta (IL-1β), and arachidonic acid were measured with bovine-specific commercial immunoassays (Wuhan Fine Biotech Co., Ltd., Wuhan, China; TNF-α, EB008; IL-1β, EB0162; arachidonic acid, EU2569). Prostaglandin E2 (PGE2) and leukotriene B4 (LTB4) were measured with high-sensitivity kits (Jianglai Biotechnology, Shanghai, China; PGE2, JL21214; LTB4, JL55244), and progesterone was measured by commercial radioimmunoassay. In the temporal replication cohort, serum 5-AED was measured by commercial radioimmunoassay (Beijing North Institute of Biological Technology, Beijing, China). Independent bovine-serum recovery, dilution-linearity, matrix-effect, and cross-reactivity experiments were not performed for the 5-AED assay, and diagnostic performance was not evaluated. The targeted measurement was therefore used to examine recurrence of the discovery-phase group pattern, not to establish analytical validation or clinical performance.

### 2.4 16S rRNA gene sequencing

Microbial DNA was extracted from fecal and rumen samples using a cetyltrimethylammonium bromide-based procedure adapted from Minas et al. (2011). Briefly, 0.5 g of sample was homogenized in 1 mL extraction buffer containing 2% cetyltrimethylammonium bromide, 100 mM Tris–HCl (pH 8.0), 20 mM EDTA (pH 8.0), and 1.4 M NaCl, followed by three freeze–thaw cycles. DNA was further purified with the QIAamp Fast DNA Stool Mini Kit (Qiagen, Hilden, Germany; catalog no. 51604). Concentration and purity were assessed with a NanoDrop One spectrophotometer and a Qubit 4 fluorometer using the Qubit double-stranded DNA high-sensitivity assay. The V3-V4 region of the bacterial 16S rRNA gene was amplified with primers 338F (5′-ACTCCTACGGGAGGCAGCA-3′) and 806R (5′-GGACTACHVGGGTWTCTAAT-3′) using Q5 High-Fidelity DNA Polymerase. Purified amplicons were prepared with the NEBNext Ultra II DNA Library Prep Kit and sequenced as 2 × 250-bp paired-end reads on an Illumina NovaSeq 6,000 platform.

Reads were demultiplexed and denoised in QIIME 2 (version 2025.10) with DADA2 (version 2025.10.0) to generate amplicon sequence variants. Chimeras were removed, and taxonomy was assigned against SILVA 138.2 (SSU Ref NR 99). Samples were rarefied to 40,000 reads for diversity analyses. Bray–Curtis dissimilarities were tested by PERMANOVA using the model Bray–Curtis dissimilarity ~ clinical group with 999 unrestricted permutations; no covariates were included in this model. A retained PERMDISP output was not available for verification. PERMANOVA is therefore interpreted as evidence of group-associated variation in Bray–Curtis structure that may reflect centroid location, within-group dispersion, or both. Ordination plots are used for visualization rather than as independent tests of separation. PICRUSt2 (version 2.6.0) was used to infer community functional potential after copy-number normalization. These predictions were used only to generate hypotheses and were not treated as measurements of microbial metabolites, intestinal permeability, or endotoxin exposure.

### 2.5 Untargeted metabolomics profiling

Metabolites were extracted from feces, rumen fluid, serum, and milk with precooled methanol/acetonitrile/water (2:2:1, vol/vol/vol). Samples were vortexed, sonicated on ice for 10 min, incubated at −20 °C for 1 h, and centrifuged at 14,000×*g* for 20 min at 4 °C. Supernatants were lyophilized and reconstituted in 100 μL of 50% methanol. A pooled quality-control sample was injected after every 10 study samples to monitor analytical stability.

Chromatography was performed on a UHPLC-Q-TOF-MS/MS system (Waters, Milford, MA, USA) with an ACQUITY UPLC HSS T3 column (1.8 μm, 2.1 × 100 mm) at 40 °C mobile phase A was water with 0.1% formic acid and mobile phase B was acetonitrile with 0.1% formic acid. Data were acquired in positive and negative electrospray-ionization modes from m/z 70 to 1,050. Progenesis QI was used for peak detection, alignment, and total-ion-current normalization. Features were annotated by accurate mass, isotope pattern, and tandem mass spectra against HMDB version 5.0 and KEGG release 110.0. Database-matched annotations were considered putative unless supported by targeted measurement. Orthogonal partial least-squares discriminant analysis was used only for exploratory feature ranking. Archived cross-validation and label-permutation diagnostics were not available for reporting; consequently, supervised score-plot separation is not used as inferential evidence. Differential features additionally required a Benjamini-Hochberg false-discovery-rate-adjusted *p* < 0.05 from a univariate group comparison, and biological interpretation emphasizes convergent targeted measurements rather than the supervised model alone.

### 2.6 TMT-based quantitative proteomics and targeted PRM assessment

Mammary proteins were extracted in 8 M urea and 50 mM Tris–HCl (pH 8.0) containing protease and phosphatase inhibitors. After centrifugation at 12,000×*g* for 10 min at 4 °C, protein concentration was determined with a bicinchoninic-acid assay. Protein (100 μg per sample) was reduced with 10 mM dithiothreitol for 1 h at 37 °C, alkylated with 30 mM iodoacetamide for 30 min in the dark, and digested overnight with sequencing-grade trypsin at an enzyme:substrate ratio of 1:50.

Discovery-cohort peptides were labeled with TMT 10plex reagents (Thermo Fisher Scientific; catalog no. 90110) and analyzed with a Q Exactive HF-X mass spectrometer coupled to an EASY-nLC 1,200 system. Spectra were searched against the *Bos taurus* UniProt reference proteome in Proteome Discoverer version 3.0. Differentially abundant proteins were defined by an absolute log2 fold change >0.26 and a Benjamini-Hochberg-adjusted *p* < 0.05. The more stringent false-discovery-rate threshold used for the volcano plots is stated in the corresponding figure caption.

Proteins selected for parallel reaction monitoring (PRM) assessment were CD44, CLDN8, FASN, ACACA, ABCG2, C5, C7, C8G, FGA, FGB, and FGG. Stable-isotope-labeled reference peptides were added at 50 fmol per sample. Scheduled PRM data were acquired on a Q Exactive HF-X instrument and processed in Skyline version 23.1. PRM results are reported as normalized log2 intensity.

### 2.7 Statistical analysis

Analyses were performed in R version 4.5.2, with the cow as the experimental unit and the discovery and temporal replication cohorts analyzed separately. Distributional assumptions were assessed with Shapiro–Wilk tests and Q-Q plots, and homogeneity of variance was evaluated before parametric testing. Normally distributed, homoscedastic outcomes were compared by one-way analysis of variance with Tukey’s *post hoc* test; other outcomes were analyzed by Kruskal–Wallis tests followed by Dunn’s multiplicity-adjusted comparisons. Parity and days in milk were included as covariates in generalized linear models for continuous outcomes when applicable. Omics-wide *p* values were adjusted by the Benjamini–Hochberg procedure (Benjamini and Hochberg, 1995). Pathway enrichment was performed in clusterProfiler version 4.14.0 with a false discovery rate <0.10 (Wu et al., 2021), and protein-interaction networks were visualized in Cytoscape version 3.10.3 (Shannon et al., 2003). Spearman correlations were used for exploratory taxon-metabolite associations. Multi-omics integration used the DIABLO framework in mixOmics version 6.35.0 (Rohart et al., 2017). Unless otherwise specified, tests were two sided and *p* < 0.05 was considered statistically significant. Summary data are presented as mean ± SD unless a figure states otherwise.

## 3 Results

### 3.1 Histological and systemic inflammatory differences among clinical groups

Histopathology and serum measurements were integrated to compare mammary and systemic phenotypes across the three clinical groups (Figure 1A). Mammary alveoli were intact in HC cows. SCM tissue showed focal inflammatory-cell infiltration and mild interalveolar edema, whereas CM tissue showed extensive loss of alveolar architecture, dense neutrophilic infiltration, liquefactive necrosis, and intravascular thrombus-like material (Figure 1B). These observations document cross-sectional differences in tissue damage among clinical phenotypes; they do not demonstrate within-animal progression.

**Figure 1:**
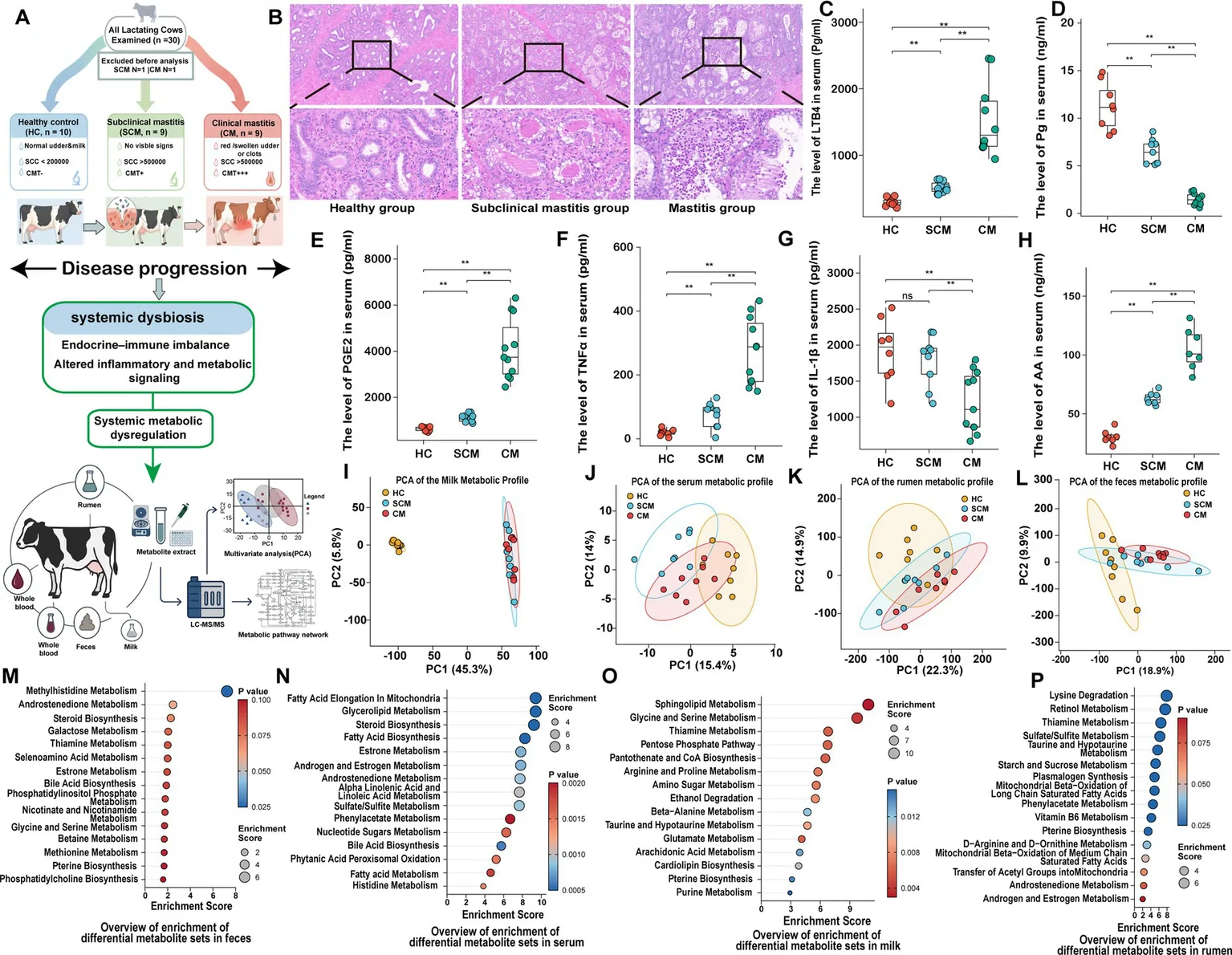
Systemic inflammatory, metabolic, and mammary histological features across mastitis clinical categories in dairy cows. **(A)** Discovery-cohort enrollment, exclusions, final analyzed sample sizes, group criteria, and sample collection. Thirty cows were initially enrolled; 28 were analyzed (HC *n* = 10, SCM *n* = 9, CM *n* = 9). **(B)** Representative hematoxylin and eosin-stained mammary sections from healthy control (HC), subclinical mastitis (SCM), and clinical mastitis (CM) cows. (C-H) Serum **(C)** leukotriene B4, **(D)** progesterone, **(E)** prostaglandin E2, **(F)** tumor necrosis factor alpha, **(G)** interleukin-1 beta, and **(H)** arachidonic acid. **(I–L)** Principal-component analysis of metabolomic profiles in **(I)** milk, **(J)** serum, **(K)** rumen fluid, and **(L)** feces. **(M–P)** Metabolite-set enrichment analyses for the corresponding matrices. Boxes show medians and interquartile ranges. *p < 0.05; **p < 0.01; ns, not significant, by one-way analysis of variance with Tukey’s test or Kruskal–Wallis test with Dunn’s test, as appropriate. Clinical categories were sampled cross-sectionally and do not imply within-animal progression.

Several circulating mediators differed among the clinical groups. LTB4, PGE2, TNF-α, and arachidonic acid were higher in SCM than in HC and were highest in CM (Figures 1C,E,F,H). Progesterone distributions also differed (Figure 1D), but reproductive stage and energy-balance covariates were not available for adequate adjustment; the result was therefore not interpreted as a mastitis-specific ordered effect. IL-1β did not differ between HC and SCM but was lower in CM than in the other groups (Figure 1G), constituting a non-monotonic pattern rather than part of a uniform inflammatory gradient. Principal-component analysis visualized variation and overlap in milk, serum, rumen-fluid, and fecal metabolomes (Figures 1I–L); no separate inferential test of separation in PCA space was performed. Enrichment analyses implicated lipid, steroid, amino acid, and carbohydrate pathways (Figures 1M–P).

### 3.2 Differences in fecal microbial community structure

Fecal richness did not differ among HC, SCM, and CM cows (Sobs index, *p* = 0.127; Figure 2C). Nonmetric multidimensional scaling visualized group-associated differences in Bray-Curtis structure (stress = 0.057), and PERMANOVA yielded *R*^2^ = 0.324 and *p* = 0.001 (Figures 2D,E). Because a corresponding multivariate-dispersion test was not retained, the PERMANOVA result may reflect differences in centroid location, within-group dispersion, or both. The data therefore support a group-associated compositional difference without evidence of a global loss of fecal richness.

**Figure 2:**
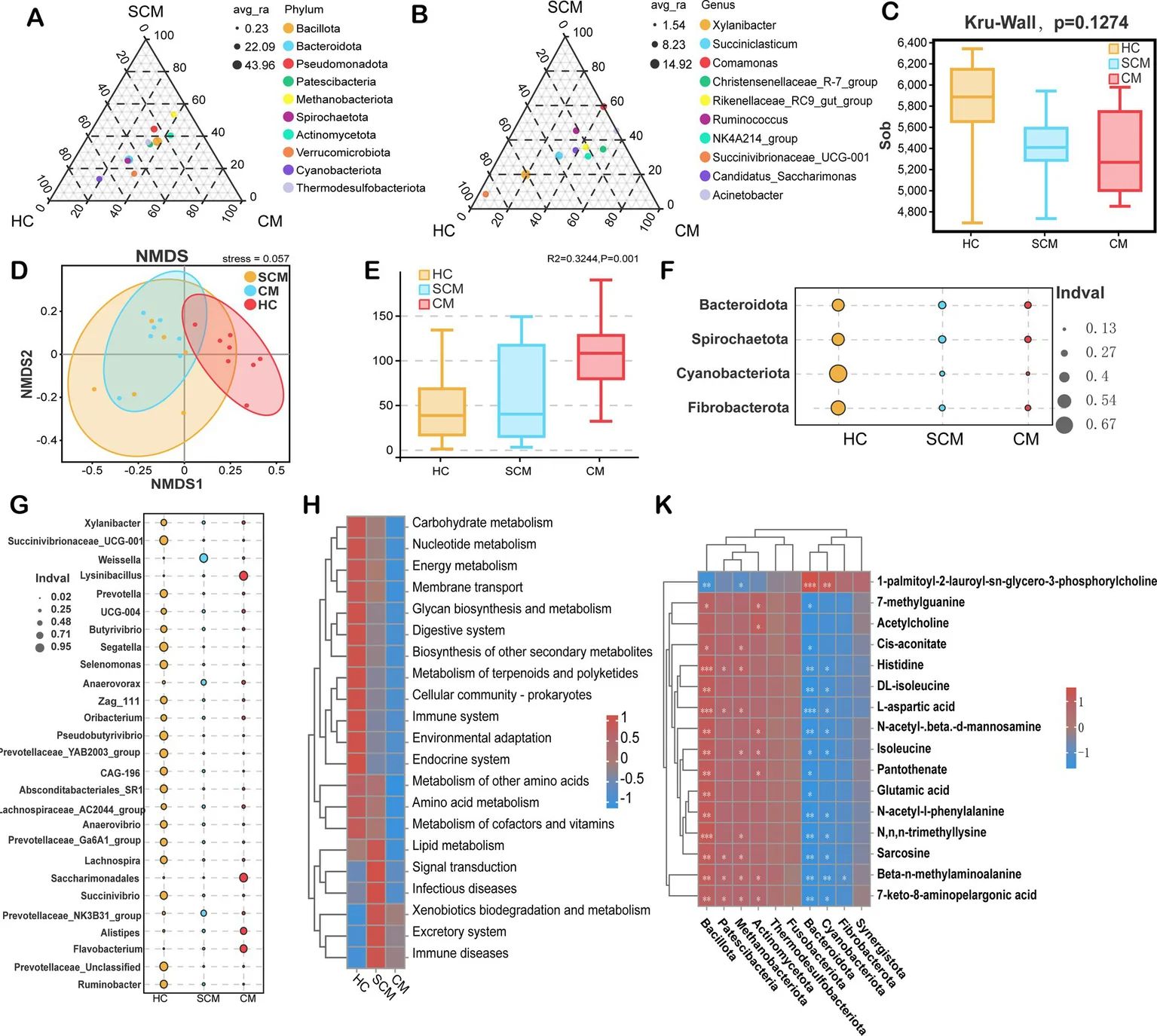
Fecal microbial community structure, predicted functional potential, and metabolite associations across mastitis clinical categories in dairy cows. (A, B) Ternary plots at **(A)** phylum and **(B)** genus levels. **(C)** Sobs richness index. **(D)** Nonmetric multidimensional scaling based on Bray-Curtis dissimilarities (stress = 0.057). **(E)** Permutational multivariate analysis of variance summary. (F, G) Indicator-value analysis at **(F)** phylum and **(G)** genus levels. **(H)** PICRUSt2-predicted KEGG functional categories. **(K)** Exploratory Spearman correlations between selected fecal metabolites and taxa. HC, healthy control; SCM, subclinical mastitis; CM, clinical mastitis. *p < 0.05; **p < 0.01; ***p < 0.001. PERMANOVA used Bray–Curtis dissimilarity ~ clinical group with 999 permutations and no covariates. A retained multivariate-dispersion test was unavailable; location, dispersion, or both may contribute to the result. The ordination is a visualization. PICRUSt2 outputs represent predicted functional potential, not measured pathway activity.

Ternary and indicator-value analyses identified taxa associated with the three clinical groups at phylum and genus levels (Figures 2A,B,F,G). Several taxa commonly annotated as short-chain-fatty-acid producers, including *Butyrivibrio* and *Pseudobutyrivibrio*, were weighted toward HC, whereas other taxa were associated with SCM or CM. PICRUSt2 predictions and exploratory taxon-metabolite correlations suggested differences in carbohydrate-, amino-acid-, and lipid-related functional potential (Figures 2H,K). These inferences do not demonstrate altered short-chain-fatty-acid production, gut permeability, or endotoxin exposure.

### 3.3 Reduced rumen richness and differences in community structure

Rumen communities also differed among groups (Figures 3A–C). Richness was lower in SCM and CM than in HC (Sobs index, *p* < 0.001; Figure 3D). Bray-Curtis ordination visualized group-associated differences (stress = 0.076), and PERMANOVA accounted for 29.5% of community variation (*p* = 0.001; Figures 3E,F). As for the fecal analysis, absence of a retained dispersion test prevents exclusive attribution of this result to centroid separation. Indicator-value analysis identified taxa associated with each clinical group (Figures 3G,H).

**Figure 3:**
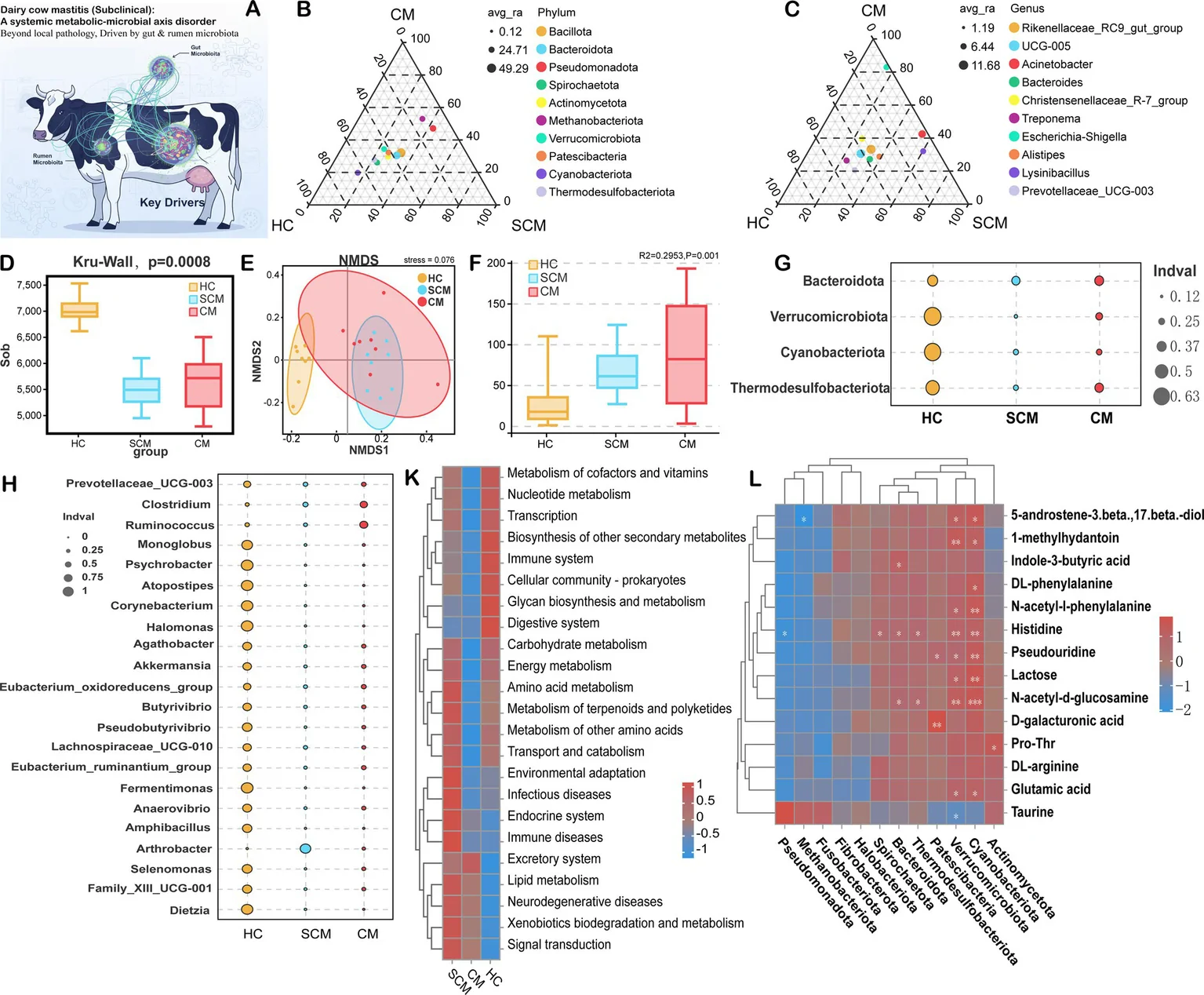
Rumen microbial community structure, predicted functional potential, and metabolite associations across mastitis clinical categories in dairy cows. **(A)** Multi-compartment study concept. **(B, C)** Ternary plots at **(B)** phylum and **(C)** genus levels. **(D)** Sobs richness index. **(E)** Nonmetric multidimensional scaling of Bray-Curtis dissimilarities (stress = 0.076). **(F)** Permutational multivariate analysis of variance summary. **(G, H)** Indicator-value analysis of rumen taxa. **(K)** PICRUSt2-predicted KEGG functional categories. **(L)** Exploratory Spearman correlations between selected rumen metabolites and taxa. HC, healthy control; SCM, subclinical mastitis; CM, clinical mastitis. *p < 0.05; **p < 0.01. PERMANOVA used Bray–Curtis dissimilarity ~ clinical group with 999 permutations and no covariates. A retained multivariate-dispersion test was unavailable; location, dispersion, or both may contribute to the result. The ordination is a visualization. PICRUSt2 outputs represent predicted functional potential, not measured pathway activity.

PICRUSt2-based predictions suggested group differences in the representation of carbohydrate- and amino-acid-metabolism functions (Figure 3K). Exploratory correlations linked selected rumen taxa with metabolites including glutamic acid, arginine, and histidine (Figure 3L). These results describe predicted functional potential and cross-sectional correlation only. They may reflect altered intake, fermentation, host inflammation, or their combination and do not establish that rumen changes initiate mastitis.

### 3.4 Multi-matrix metabolomics prioritizes serum 5-AED

Untargeted metabolomics across rumen fluid, feces, serum, and milk identified extensive but compartment-specific differences among HC, SCM, and CM cows (Figures 4A–E). The overlap analysis showed both shared and matrix-restricted differential features (Figure 4G), indicating concurrent differences across compartments rather than uniform movement of the same metabolites between organs.

**Figure 4:**
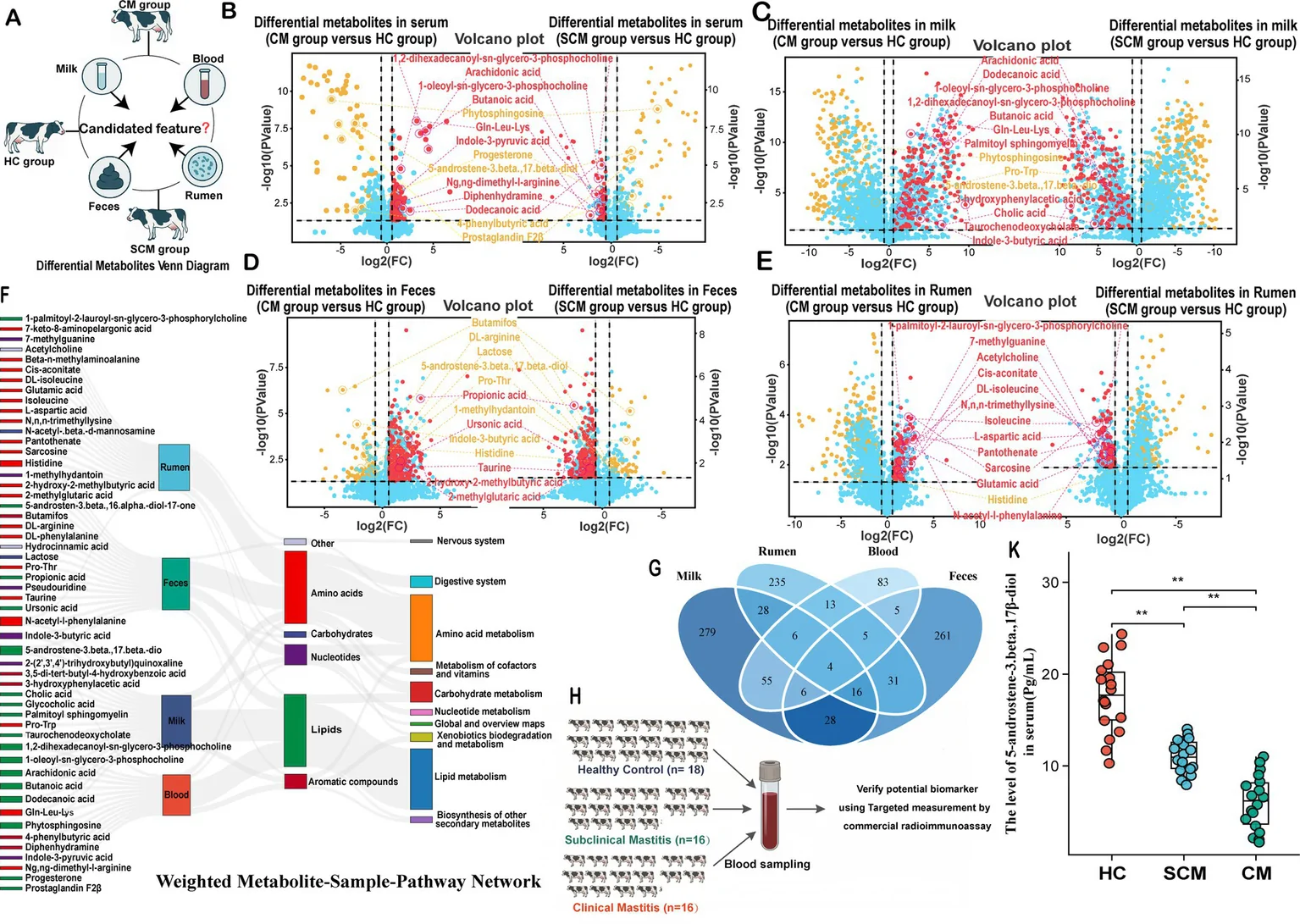
Multi-compartment metabolomics and targeted measurement of 5-androstene-3β,17β-diol across mastitis clinical categories in dairy cows. **(A)** Cross-matrix candidate-feature screening strategy in the discovery cohort. **(B–E)** Volcano plots for **(B)** serum, **(C)** milk, **(D)** feces, and **(E)** rumen fluid; variable-importance-in-projection score >1 and false-discovery-rate-adjusted *p* < 0.05. **(F)** Weighted metabolite-sample-pathway network. **(G)** Overlap of differential metabolite features across matrices. **(H)** Same-herd temporal replication cohort: 54 cows initially enrolled and 50 included in the final analysis (HC *n* = 18, SCM *n* = 16, CM *n* = 16). **(K)** Targeted serum 5-androstene-3β,17β-diol measurement by commercial radioimmunoassay in the temporal replication cohort. HC, healthy control; SCM, subclinical mastitis; CM, clinical mastitis. **p < 0.01. The temporal replication cohort was recruited from the same herd and does not constitute external validation.

Serum and milk differences were enriched for lipid-related metabolites, including arachidonic acid, phytosphingosine, and phosphatidylcholines, whereas fecal and rumen profiles emphasized amino acid, carbohydrate, and cofactor metabolism (Figures 4B–4F). The weighted metabolite-sample-pathway network summarized these compartmental relationships but did not measure metabolic flux or metabolite transport between organs.

5-AED was among the candidates identified in discovery-cohort untargeted serum metabolomics (Figures 4A–G). Targeted radioimmunoassay in the later same-herd cohort showed the highest serum 5-AED distribution in HC, an intermediate distribution in SCM, and the lowest distribution in CM (*p* < 0.01; Figures 4H,K). This recurrence across sampling periods and analytical modes supports an association with clinical/SCC phenotype. It does not establish within-animal decline, diagnostic accuracy, prognosis, mastitis specificity, or external-herd reproducibility.

### 3.5 Mammary barrier, lipid-metabolic, and innate immune proteins differ among clinical groups

Principal-component analysis visualized the distribution of the mammary TMT proteomic profiles across the three groups (Figure 5A); no separate inferential test of separation in PCA space was performed. Differential-abundance analysis identified clinical-group-associated differences in bulk-tissue protein abundance (Figures 5B–E). CLDN8, a component of epithelial tight junctions, and ITGB6, an integrin involved in epithelial–matrix interactions, were less abundant in mastitis tissue. Pathway and network analyses showed enrichment of NOD-like receptor signaling, complement and coagulation cascades, and a neutrophil-extracellular-trap-associated annotation (Figures 5F–J). These are tissue-level abundance and pathway-annotation results; they do not establish cell-intrinsic regulation, receptor activation, or extracellular-trap formation.

**Figure 5:**
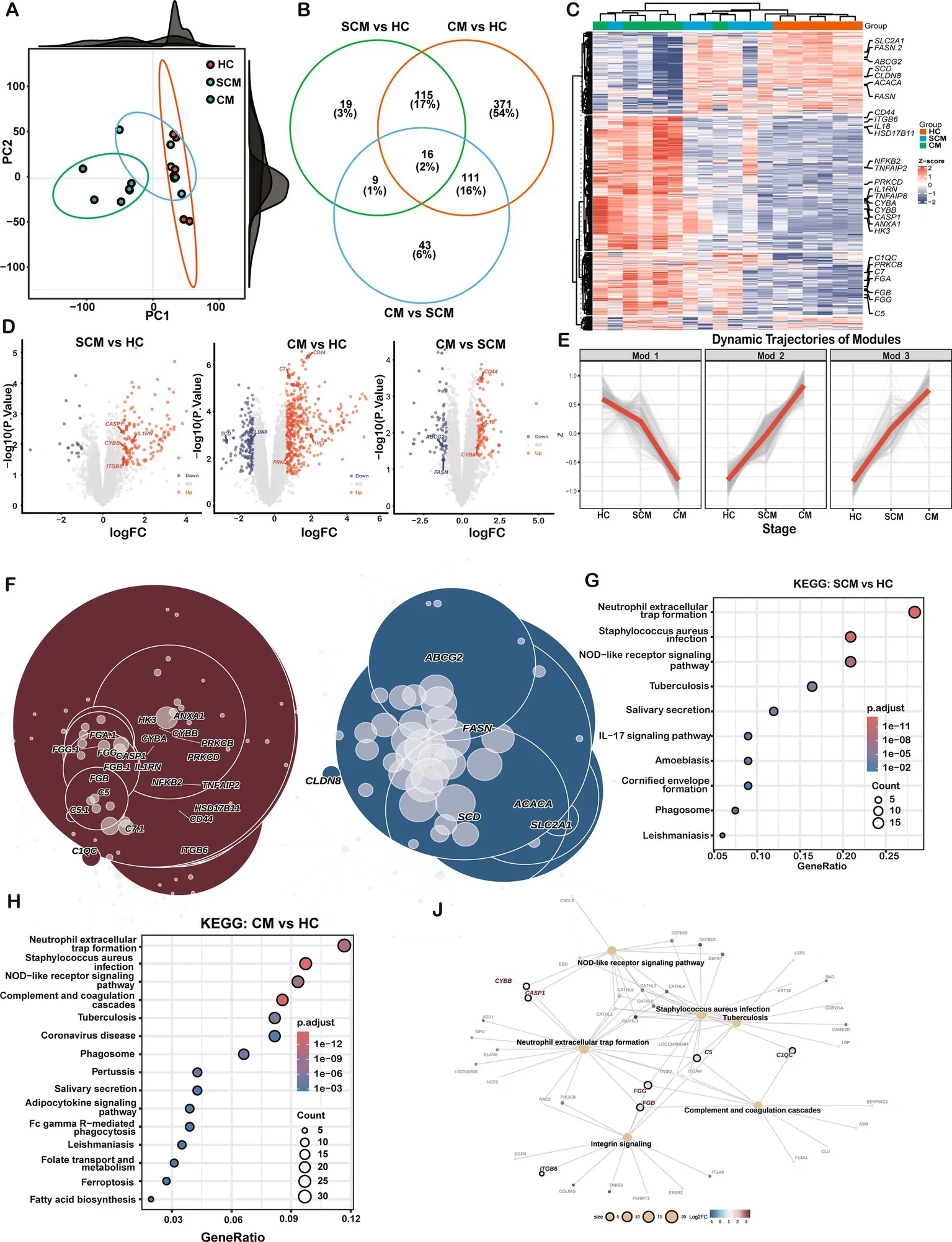
Mammary tissue proteomic differences across mastitis clinical categories in dairy cows. The TMT analysis used discovery-cohort mammary tissue (HC *n* = 3, SCM *n* = 3, CM *n* = 3). **(A)** Principal-component analysis used for visualization of tandem-mass-tag proteomic profiles. **(B)** Overlap of differentially abundant proteins. **(C)** Hierarchical clustering. **(D)** Volcano plots for subclinical mastitis (SCM) versus healthy control (HC), clinical mastitis (CM) versus HC, and CM versus SCM; false discovery rate <0.01 and absolute log2 fold change >0.26 for the displayed subset. **(E)** Dynamic protein-abundance trajectories. **(F)** Protein-interaction network. (G, H) KEGG pathway enrichment for **(G)** SCM versus HC and **(H)** CM versus HC. **(J)** Gene-concept network linking selected proteins with enriched pathways. The neutrophil extracellular trap term is a pathway annotation and is not direct evidence of NET formation.

Proteins involved in *de novo* lipid synthesis, including FASN, ACACA, and SCD, were also less abundant, together with the transporter ABCG2 (Figures 5C,F). These differences were most pronounced in CM and coincided with loss of alveolar structure. Because bulk mammary tissue contains epithelial, stromal, vascular, and infiltrating immune cells, the observed abundances may reflect both altered cellular programs and changes in tissue composition. The data therefore support concurrent tissue remodeling but do not establish that reduced epithelial lipogenesis causes barrier disruption.

### 3.6 Targeted verification of selected mammary proteins

PRM was used as an orthogonal targeted assessment in a discovery-cohort mammary-tissue subset (HC *n* = 3, SCM *n* = 3, CM *n* = 3; Figures 6A–O). CD44, complement components C5, C7, and C8G, and fibrinogen chains FGA, FGB, and FGG were more abundant in mastitis tissue, whereas CLDN8, FASN, ACACA, and ABCG2 were less abundant. The largest contrasts generally involved CM versus HC; several SCM-versus-CM comparisons were not significant. PRM was not performed in the later serum 5-AED cohort and does not constitute independent-cohort or independent-herd validation.

**Figure 6:**
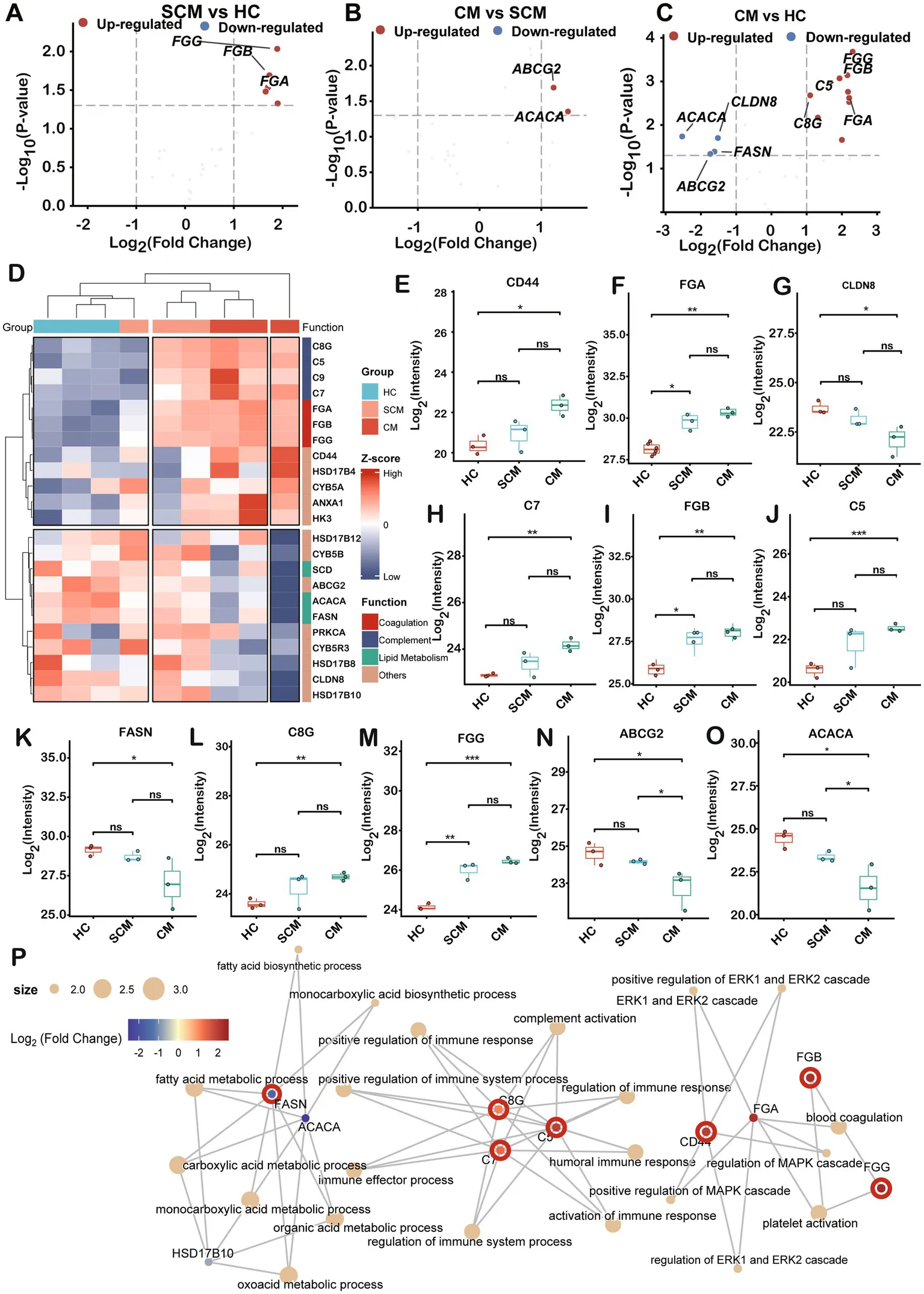
Parallel-reaction-monitoring assessment of mammary barrier-, lipid-metabolic-, complement-, and coagulation-related proteins across mastitis clinical categories in dairy cows. PRM used discovery-cohort mammary tissue (HC *n* = 3, SCM *n* = 3, CM *n* = 3) and was not performed in the temporal replication cohort. **(A–C)** Volcano plots for **(A)** subclinical mastitis (SCM) versus healthy control (HC), **(B)** clinical mastitis **(CM)** versus SCM, and **(C)** CM versus HC. **(D)** Heatmap of selected discovery-proteomics candidates, including hydroxysteroid 17β-dehydrogenases. **(E–O)** Normalized log2 intensity of **(E)** CD44, **(F)** FGA, **(G)** CLDN8, **(H)** C7, **(I)** FGB, **(J)** C5, **(K)** FASN, **(L)** C8G, **(M)** FGG, **(N)** ABCG2, and **(O)** ACACA. **(P)** Functional network of selected proteins and enriched biological processes. *p < 0.05; **p < 0.01; ***p < 0.001; ns, not significant. Normally distributed, homoscedastic outcomes were analyzed by one-way analysis of variance with Tukey’s *post hoc* test; other outcomes were analyzed by Kruskal–Wallis tests with Dunn’s multiplicity-adjusted comparisons.

Functional-network analysis connected selected complement and fibrinogen proteins with annotations related to humoral immunity, coagulation, platelet activation, and mitogen-activated protein kinase signaling (Figure 6P). Together with histological thrombus-like material, the findings suggest local thromboinflammatory remodeling. Direct measurements of extracellular traps, platelet activation, and fibrin deposition would be required before mammary immunothrombosis could be established mechanistically.

### 3.7 Mammary HSD17B abundance and circulating 5-AED

Cross-matrix screening and targeted serum measurement identified lower circulating 5-AED concentrations across mastitis clinical categories. Because 5-AED has shown anti-inflammatory activity in other experimental systems, the association may reflect altered endogenous steroid regulation. The present study did not measure 5-AED synthesis, clearance, receptor activity, or nuclear factor kappa B phosphorylation and therefore cannot determine whether 5-AED is a driver, consequence, or correlate of inflammation.

Examination of the mammary proteome showed clinical-group-associated abundance patterns for HSD17B4, HSD17B10, and HSD17B12, with HSD17B10 and HSD17B12 less abundant in CM tissue (Figure 6D). These findings generate a systems-level hypothesis connecting steroid-redox metabolism with the circulating 5-AED pattern. Hydroxysteroid 17β-dehydrogenases catalyze bidirectional, substrate-specific reactions, however, and neither enzyme activity nor a direct biochemical reaction involving 5-AED was tested. The HSD17B-5-AED relationship is therefore a mechanistic hypothesis, not an established pathway.

## 4 Discussion

This study identifies concurrent cross-compartment features associated with bovine mastitis clinical status. Three observations were reproduced or converged across analytical layers: gastrointestinal microbial communities differed by clinical group; circulating 5-AED concentrations showed the same group ordering in a later, same-herd cohort analyzed separately; and mammary tissue showed lower abundance of barrier- and lipid-metabolic proteins alongside complement-coagulation enrichment. These data extend the phenotypic description beyond the mammary gland while preserving an essential distinction: they demonstrate covariance across compartments, not temporal progression or a directional gut-to-mammary mechanism.

The targeted measurements served different purposes and used different cohorts. PRM provided an orthogonal assessment of selected mammary proteins in a discovery-cohort tissue subset, whereas radioimmunoassay tested the serum 5-AED pattern in the later same-herd cohort. In contrast, 16S-derived functional predictions, database-matched metabolite annotations, enrichment analyses, and cross-omics correlations were considered exploratory. None of these analyses establishes molecular transport, temporal order, or causation.

The fecal and rumen findings are compatible with hypotheses raised by experimental work on the gut/rumen-mammary axis (Hu et al., 2020, 2024). Putative short-chain-fatty-acid-producing taxa were redistributed, rumen richness was lower, and 16S-based predictions indicated altered functional potential. Butyrate can support epithelial energy metabolism and modulate junctional and immune programs (Koh et al., 2016; Liu et al., 2018; Mohamed Elfadil et al., 2023; Singh et al., 2023). In the present study, however, short-chain fatty acids, intestinal permeability, and circulating endotoxin were not measured. The data therefore do not show reduced short-chain-fatty-acid production, barrier failure, or endotoxin exposure. Group-level dry-matter intake was lower in SCM and CM than in HC (19.1 and 17.8 versus 23.8 kg/cow/day), while milk yield was also lower (28.4 and 23.4 versus 38.2 kg/cow/day). These contemporaneous differences could contribute to the microbial and metabolomic profiles and may themselves be consequences of illness. Reverse causality and nutritional confounding therefore remain plausible.

Metabolomics added a second layer by showing that gastrointestinal matrices were dominated by amino acid and carbohydrate changes, whereas serum and milk were enriched for lipid remodeling. Branched-chain amino acids can influence immune-cell metabolism through mammalian target of rapamycin, general control nonderepressible 2, and mitochondrial transamination pathways (Ananieva-Stoyanova et al., 2021; Yang et al., 2023; Yao et al., 2023), while short-chain fatty acids provide immunoregulatory signals (Parada Venegas et al., 2019). The present profiles are consistent with a redistribution of immune-metabolic substrates, but untargeted abundance data cannot resolve production, consumption, or inter-organ flux. Stable-isotope tracing will be needed to test whether any metabolite moves causally from the gastrointestinal tract to the mammary gland.

The most distinctive circulating candidate was 5-AED. Its discovery-phase metabolomic pattern and targeted recurrence in the later cohort support an association with clinical/SCC phenotype, not diagnostic performance, prognosis, or mastitis specificity. Progesterone also differed among groups. Available records showed lower group-level milk yield and dry-matter intake in SCM and CM, whereas mean body-condition scores were similar (HC 3.18, SCM 3.25, and CM 3.28). Estrous-cycle stage and pregnancy status were unavailable, and individual-level adjustment was not possible. The progesterone result may therefore reflect reproductive or systemic metabolic physiology and is not used to support the proposed 5-AED relationship. Although 5-AED can suppress inflammatory signaling in experimental models (Abbas and Hassan, 2022), its biological activity in bovine mastitis and independence from endocrine and inflammatory covariates remain unknown. Concurrent mammary HSD17B abundance patterns generate a steroid-redox hypothesis but do not identify the source of circulating 5-AED. Enzyme assays, isotope-labeled steroid tracing, and tissue-specific perturbation are required before assigning any HSD17B enzyme upstream of 5-AED.

The lower serum IL-1β concentration in CM should not be interpreted as weaker mammary inflammation. A single circulating measurement can differ from the local mammary response because cytokine release is transient, compartmentalized, and influenced by disease timing, receptor binding, clearance, and systemic immune regulation. Milk and tissue IL-1β and serial serum measurements were not obtained, so the present study cannot distinguish among these explanations.

Mammary proteomics revealed concurrent tissue-level reductions in barrier-associated and lipid-synthetic proteins. Lower CLDN8 is consistent with, but does not directly demonstrate, weakening of the blood–milk barrier; lower FASN, ACACA, SCD, and ABCG2 likewise indicates altered tissue abundance rather than epithelial-cell-specific regulation. Membrane lipid composition can influence receptor organization and inflammatory signaling (Płóciennikowska et al., 2015; Varshney et al., 2016), providing a plausible connection between metabolic and barrier remodeling. Bulk-tissue composition, including epithelial loss and inflammatory-cell infiltration, is an important alternative explanation. The appropriate conclusion is concurrent remodeling, not a demonstrated FASN-ACACA-driven barrier mechanism.

Complement and coagulation formed the clearest targeted mammary inflammatory module. Increased C5, C7, C8G, and fibrinogen chains converged with histological thrombus-like material and enrichment of a neutrophil-extracellular-trap-associated pathway annotation. Extracellular traps can provide procoagulant scaffolds and amplify tissue injury (Fuchs et al., 2010; Papayannopoulos, 2018; Stark and Massberg, 2021), and this link is relevant to cattle (Xie et al., 2021). Recent work further connects neutrophil membrane remodeling, complement-mediated injury, and procoagulant platelets with thromboinflammation (Colicchia et al., 2022; Liu W. et al., 2026; Liu X. et al., 2026; Noone et al., 2024; Zhu et al., 2023). Extracellular traps and platelet activity were not measured here, so these findings suggest NET-related processes but do not demonstrate NET formation or mammary immunothrombosis.

Several limitations define the scope of inference. The clinical groups were sampled cross-sectionally and were defined by clinical examination, quarter-level California Mastitis Test, and SCC rather than pathogen identification. Enrollment from one herd limits generalizability, and the later cohort provides temporal replication within that herd rather than external validation. Milk yield, dry-matter intake, and body-condition score were available only as group means; estrous-cycle stage and pregnancy status were unavailable. These data document production and intake differences but do not permit individual-level adjustment or determine whether reduced intake preceded or followed mastitis. Quarter selection and the changing cellular composition of bulk mammary tissue may contribute to the proteomic contrasts. The study did not directly measure intestinal permeability, endotoxemia, short-chain fatty acids, extracellular traps, enzyme activity, or 5-AED signaling. PERMDISP output and complete OPLS-DA cross-validation and permutation diagnostics were unavailable, so ordination and supervised findings are interpreted conservatively. Finally, diagnostic and prognostic performance for 5-AED was not assessed. Longitudinal, pathogen-stratified, intake-controlled studies in independent herds, together with independent assay validation, are required.

In conclusion, clinical/SCC-defined mastitis phenotypes were associated with concurrent differences in gastrointestinal microbial communities, circulating metabolites, and bulk mammary-tissue proteins. Lower circulating 5-AED in the later same-herd cohort was consistent with the discovery metabolomic pattern, but its diagnostic, prognostic, and mechanistic significance remains undetermined. The accompanying HSD17B abundance patterns generate a testable hypothesis rather than an established pathway. Longitudinal studies in independent herds are needed to determine temporal order, control reproductive and nutritional covariates, and evaluate clinical utility.

## Data Availability

The original contributions presented in the study are publicly available. This data can be found here: The raw 16S rRNA gene-sequencing data have been deposited in the NCBI Sequence Read Archive under BioProject accession PRJNA1523039 (https://www.ncbi.nlm.nih.gov/sra/PRJNA1523039). The raw proteomics data are available through the ProteomeXchangeConsortium under dataset identifier PXD083629 (https://proteomecentral.proteomexchange.org/cgi/GetDataset?ID=PXD083629).
